# The impact of China pilot carbon market policy on electricity carbon emissions

**DOI:** 10.1038/s41598-025-00975-7

**Published:** 2025-05-12

**Authors:** Zelong Zhang, Yanli Xiao, Kun Zhang, Mengyuan Tang, Ting Ma

**Affiliations:** State Grid Ningxia Electric Power Co, Economic and Technical Research Institute, Yinchuan, 750000 China

**Keywords:** Electric power industry, Carbon market, Carbon emission reduction, Multi-period difference-in-differences model, Spatial Durbin model, Environmental sciences, Environmental social sciences, Energy science and technology

## Abstract

The electric power industry is the pillar of the national economy but also the largest carbon emission sector in China, facing great pressure to reduce emissions. Existing research often lacks the analysis of the carbon market on electricity carbon emission reduction. Based on the panel data of 30 provinces (cities) in China from 2003 to 2020, we combine the multi-period difference-in-differences model with the spatial Durbin model to explore the effectiveness of the pilot carbon market policy on electricity carbon emission reduction, the mechanism of its role, and its spatial spillover effect. The results show that: (1) The carbon market policy in the electric power industry can effectively play the carbon emission reduction effect, but its spatial spillover effect exists in the carbon emission transfer phenomenon to weaken the overall emission reduction effect. (2) The pilot carbon market policy mainly promotes carbon emission reduction in the power industry by strengthening government intervention and reducing power energy consumption, and the role of R&D innovation intensity has not yet been substantially realized. (3) The impact of pilot carbon market policies on electricity carbon emission reduction varies according to the degree of emphasis on environmental governance and regional features. The conclusion provides feasible suggestions for policymakers to strengthen the mechanism design of the carbon market in the power industry, which is of great significance for realizing the goal of “double carbon.”

## Introduction

Carbon emissions are one of the main drivers of global climate change, leading to the intensification of the greenhouse effect, sea level rise, and the frequency of extreme weather events, which have seriously threatened the sustainable development of human beings^1^. The international community has taken a series of measures to address carbon emissions, including international agreements such as the Paris Agreement, which aims to reduce greenhouse gas (GHG) emissions and protect the Earth’s environment. Countries are also developing and implementing their own emission reduction plans, such as China’s “Carbon Peak, Carbon Neutral” action plan and the U.S. Clean Power Plan. According to the International Energy Agency (IEA) and the Global Carbon Project, China’s carbon emissions account for about 28% of the world’s total emissions, making it the world’s largest CO_2_ emitter^2^. For this reason, the Chinese government has proposed to actively realize “carbon neutrality” and “carbon peaking”. In order to realize the goal of “double carbon,” in 2013, China’s carbon emissions trading market began to carry out online trading in seven pilot cities one after another, Beijing, Shanghai, Tianjin, and Guangdong took the lead. In 2014, Hubei and Chongqing began trading activities, and in early 2017, Fujian began carbon trading. In July 2021, the National Carbon Emission Trading Market was launched for online trading, becoming the largest carbon trading market in the world in terms of the scale of greenhouse gas emissions covered^3^, but compared with the EU ETS and the Regional Greenhouse Gas Initiative (RGGI), China’s carbon market policy is still insufficient, and its long-term policy effect is uncertain^4^.

During the “14th Five-Year Plan” period, China further proposed to accelerate the construction of a new type of power system, strengthen the clean and efficient use of coal, and promote the low-carbon transformation and construction of coal power. The report of the 20th Party Congress pointed out that we should actively and steadily promote carbon peak and carbon neutrality and strengthen the carbon emission rights market trading system. The development of the power industry is related to the national economy and people’s livelihood, but it is also the main source industry of carbon emissions. At this stage, carbon emissions from the power industry account for more than 40% of China’s total CO_2_ emissions^5^, and promoting carbon emission reduction in the power industry is crucial for realizing the “dual-carbon” goal.

Currently, the main position of thermal power generation remains unshaken, and it is the “ballast” of the power system^6^. China’s coal power industry is under the double pressure of “clean and efficient utilization” and “low-carbon transformation,” and at the same time, under the superimposed cost of carbon, the power industry is facing the problem of revenue^7^. There is limited literature examining the impact of carbon markets on the power industry. Specifically, it remains unclear whether the implementation of pilot carbon markets has generated emission reduction effects on carbon emissions in the power sector and whether such effects exhibit policy lag. Furthermore, in the context of balancing the trilemma of ensuring energy supply, achieving carbon reduction, and maintaining economic efficiency, how carbon markets can effectively reconcile these competing priorities requires further exploration. Answering these questions is conducive to providing a scientific basis for improving China’s carbon market policy, promoting the low-carbon transformation of the power industry, facilitating high-quality and sustainable economic development, and is of great significance to the realization of the dual-carbon goal.

This paper evaluates the effect of the carbon trading market on carbon emissions in the power sector and explores the mechanism of its effect on the power sector, the objectives of this paper are: (1) to make up for the shortcomings of the existing studies on the impact of the carbon market on different industries and provide new insights into the synergies between the carbon market and the electricity market to optimize the design of the carbon market and contribute to the smooth expansion of the national carbon market; (2) Through mechanism analysis, we break through the limitation of “focusing on the total amount but not on the structure” of carbon market research and clarify the main channels of carbon market in the electric power industry, based on which we can provide stage-by-stage optimization strategies for the improvement of the policy and provide references for the evaluation of the industry-level policy.

## Literature review

China’s carbon emissions trading market through market-based means to achieve emission reduction targets, although in practice time lags behind the European Union, but the development of faster, with greater potential^8^. In 2013, 2014, Beijing and other places in China’s carbon market policy landed, as an opportunity for a series of articles on the emission reduction effect of the carbon trading policy has been extensively studied. The existing literature on the assessment of the effectiveness of the pilot carbon market policy mainly comes from the quantitative analysis of environmental effects and economic effects. In the environmental emission reduction effect, scholars have found that the carbon market significantly reduces carbon emissions^9^ and carbon intensity^10^ in the pilot region, the pilot region than the non-pilot region decreased by more than 10%, but found that the intensity of administrative intervention on the carbon market emission reduction effect has a certain enhancement effect, which reflects China’s carbon market policy market mechanism is not yet fully mature^11^. In synergistic emission reduction effects, the study found that the pilot carbon market policy is not only able to significantly reduce regional carbon dioxide emissions, but also significantly reduce regional sulfur dioxide, industrial wastewater, industrial solid waste emissions, but the carbon market in carbon reduction and pollution reduction intensity is a difference, the carbon market on the carbon reduction of the impact of the increase year by year, pollution reduction is a short-term effect^12^. Scholars have also applied spatial Durbin modeling to analyze the policy’s spatial spillover effect. The study found that carbon leakage between the pilot cities may be more likely to occur than between non-pilot cities, and this spatial externality may weaken the overall effect of emission reduction^13^. For the spillover effect of the policy, scholars have not reached a unanimous conclusion. Some scholars believe that the carbon trading pilot policy effectively suppresses carbon emissions in the pilot areas on the basis of the spillover effect of the policy to help inhibit carbon emissions in neighboring areas, and the direct emission reduction effect is stronger than the indirect emission reduction effect^14^. However, some scholars found that the implementation of carbon emission reduction policy has some emission transfer effect between neighboring regions, and the spatial spillover effect of the policy has not been fully released^15^. In terms of economic effects, scholars have found that the pilot carbon market policy has certain impacts on enterprise performance^16^ and employment effects^17^.

At present, scholars’ research on carbon emissions from the electric power system is mostly concerned with the influencing factors of low-carbon transition, carbon emission paths, etc^18^. , and the research methods mostly use spatial econometric modeling and LMDI decomposition. According to the literature, the influencing factors of electric power carbon emissions mainly include GDP per capita, electric power production structure, energy efficiency, coal consumption of power generation, technological progress, population size, industrial structure, etc^19–21^. However, based on the different research regions, methodologies, data sources, calibers, etc., the degree of influence and direction of electric power carbon emissions by various types of effects may be different.

In summary, existing studies on pilot carbon market policies have generated substantial progress. Scholars have conducted extensive and in-depth investigations into the overall emission reduction effects of these markets^22,23^. The carbon trading policy on the environment has a positive impact, but the economic effects of the carbon trading policy and the spatial spillover effects need to be more comprehensive and in-depth. These works provide more important literature support for the research of this paper, but the existing research still has some shortcomings. Firstly, the existing research on carbon market policy mostly focuses on the emission reduction effect of the overall economy or industrial sector, but the targeted analysis of the electric power industry is insufficient. Secondly, due to its high carbon dependency and technological complexity, the carbon emission reduction mechanism of the power sector may be significantly different from that of other industries, but the existing literature lacks an in-depth exploration of the dynamic relationship between the internal emission reduction pathway of the power sector and carbon market policies. In terms of policy assessment methods, the existing literature mostly adopts the traditional double-difference model and robustness test, which is not comprehensive enough to explore the endogeneity of policies.

**Fig. 1:**
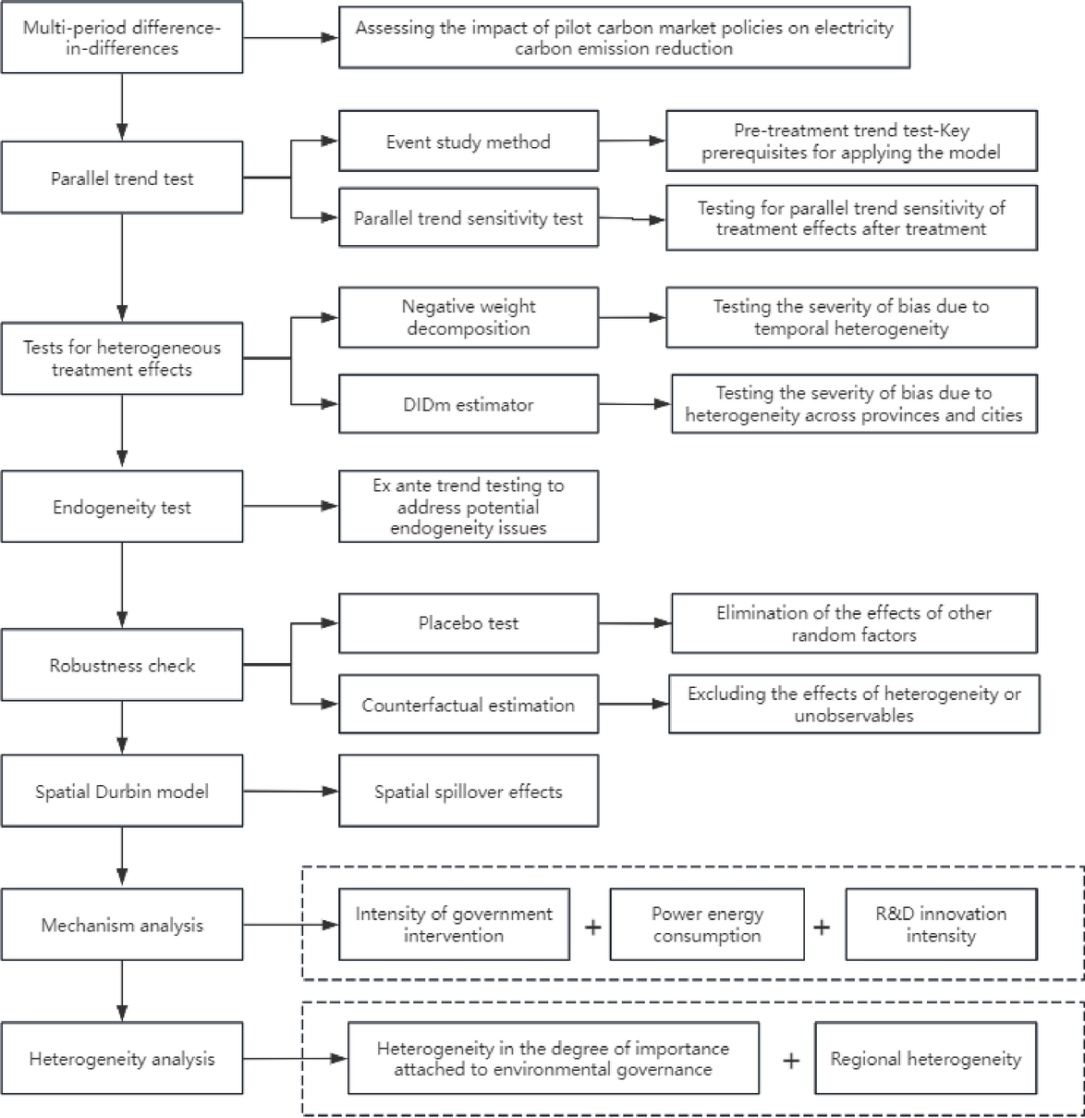
Framework of analysis.

The marginal contribution of this paper may lie in the following aspects: (1) using the panel data of 30 provinces and cities in China from 2003 to 2020, the multi-period double-difference model is used to explore the impact effects of the pilot carbon market policy on the power industry, which broadens the perspective and enriches the content of the existing research on carbon trading policy; (2) Combining the latest theoretical and econometric literature, we adopt various testing methods, such as parallel trend sensitivity and heterogeneity treatment effect test, to overcome the bias caused by the traditional model; (3) Focusing on the internal structure of the electric power industry, we reveal the mechanism by which China’s carbon emissions trading pilot affects the electric power industry, including supplementing the microtheoretical basis of the transformation of the electric power system under the goal of “dual-carbon”. (4) Apply the spatial Durbin model to examine the spatial spillover effect of the pilot carbon market and assess the impact of the policy in a more rational way. The specific analytical idea of this study is shown in Fig. 1.

## Theoretical analysis and research hypothesis

### Electricity carbon emission reduction effects of carbon markets

Carbon emissions trading policy (ETS) has been widely recognized as a market-based policy instrument with effectiveness and adaptability^24–26^, and the carbon emissions trading market serves as a market mechanism by pricing carbon emission rights to incentivize emission reduction behavior in the power sector. Its core lies in treating carbon emission rights as a tradable commodity, which enterprises buy and sell based on the difference between their own carbon emissions and their allocated carbon allowances. The implementation of the carbon market al.lows over-emitting power companies to purchase additional carbon emission rights, increasing the cost of power generation and shrinking profits^27^. In addition, the carbon market can also guide enterprises to optimize their production structure through the quota allocation mechanism, prompting high-carbon-emitting enterprises to transform to low-carbon or zero-carbon technologies^28,29^. Accordingly, the following hypotheses are proposed on the basis of the theoretical model:

#### Hypothesis 1

The pilot carbon market policy is conducive to promoting the carbon emission reduction effect of electricity.

### The mechanism of carbon market electricity emission reduction

The carbon market is essentially a market-based tool to guide emission reduction through price signals, but its effectiveness depends on perfect market design, sufficient information transparency, and a reasonable regulatory framework^30^. However, China’s carbon market is still in the primary stage; there are problems such as administrative allocation of quotas, sharp price fluctuations, limited coverage, etc^8^. ; the government needs to make up for the short-term market failure through price regulation, dynamic quota adjustment, and other administrative interventions to help the long-term emission reduction effect. When the carbon market mechanism has not yet been established completely and effectively, and under the pressure of carbon emission reduction, the local government has the incentive to strengthen the traditional administrative intervention-type tools for carbon emission reduction^11^. Accordingly, the following hypotheses are proposed on the basis of the theoretical model:

#### Hypothesis 2

Pilot carbon market promotes electricity carbon emission reduction by strengthening government intervention.

The carbon emissions trading policy raises the cost of carbon emissions for enterprises by pricing carbon emissions. In order to reduce the cost of carbon emissions, enterprises may adopt more efficient power generation equipment and technology, improve the efficiency of coal-fired power generation, and optimize the production process, thus reducing the energy consumption per unit of output. With the increase of carbon cost, the proportion of carbon cost of coal-fired units to the cost of power generation climbs gradually, which prompts enterprises to reduce carbon emissions through energy-saving renovation and carbon reduction and abatement measures to reduce the compliance cost and impact on generating units. Accordingly, the following hypotheses are proposed on the basis of the theoretical model:

#### Hypothesis 3

Reducing power energy consumption is one of the important ways to promote power carbon emission reduction in the pilot carbon market.

Technological innovation has become an important driving force for the green transformation of energy and power systems^31^. With the combination of technological innovation and carbon market policies, enterprises with a high technological level and low abatement costs can gain revenue by selling excess allowances, while enterprises with a low technological level need to pay extra carbon emission costs, which will push them to increase technological innovation and abatement investment. Enterprises rationally^32^will invest more R&D funds in R&D or improvement of power generation technology, which achieves the emission reduction effect by improving the efficiency of coal power units, incentivizing the development of CCUS technology, and promoting the substitution of renewable energy for fossil energy power generation^33^. Accordingly, the following hypotheses are proposed on the basis of the theoretical model:

#### Hypothesis 4

The pilot carbon market promotes electricity carbon emission reduction by strengthening the intensity of R&D innovation.

## Method and data

### Model setting

#### DID model

As a quasi-experimental research design, the DID model can estimate the policy effect more accurately by setting up experimental and control groups, which can avoid the endogeneity problem to a large extent. Due to the gradual progress of the pilot provinces, the time of trading in each pilot province is not consistent. In order to accurately identify the policy effect in multiple periods, this paper adopts a multi-temporal double-difference model to assess the carbon emission reduction effect of the implementation of the carbon market policy on electric power. The DID model is set as in Eq. (1).

1 $$\:{y}_{i,t}=\alpha\:+\beta\:DI{D}_{it}+\gamma\:\sum\:{X}_{it}+{\mu\:}_{i}+{\lambda\:}_{t}+{\epsilon\:}_{it}$$

where the explanatory variable y_*it*_ is the electricity carbon emissions of province *i* in year *t*, DID_*it*_ reflects whether or not province *i* implements a carbon trading policy in year *t*. If province *i* is set up as a carbon trading pilot in year *t*, DID_*it*_ takes the value of 1; otherwise, it takes the value of 0, µ_*i*_ denotes the province fixed effect, λ_*t*_ is the year fixed effect, and X_*it*_ is a series of control variables affecting the electricity carbon emissions of province *i* in year *t.* X_*it*_ controls a series of regional characteristics that may have an impact on local electricity carbon emissions, and ε_*it*_ is a random perturbation term affecting province carbon emissions.

#### Spatial Durbin model

It has been found that there is a spatial correlation feature between carbon emissions^34^ and environmental regulation^35^, and whether there is a spillover effect of the pilot carbon market policy is also a part of the policy effect, and ignoring the spillover effect of the pilot carbon market policy during the assessment process of the policy carbon emission reduction may lead to biased results of the policy assessment. To solve this problem, the spatial Durbin model is used for the test study, and the pilot carbon market policy dummy variable (DID) is included in the model, and the model is adjusted as follows Eq. (2):

2 $$\:{y}_{i,t}=\alpha\:+\rho\:W{y}_{i,t}+\beta\:{DID}_{i,t}+\phi\:W{DID}_{i,t}+\gamma\:\sum\:{X}_{i,t}+\delta\:W\sum\:{X}_{i,t}+{\mu\:}_{i}+{\lambda\:}_{t}+{\epsilon\:}_{i,t}$$

where W is the spatial weight matrix, $\:\rho\:\:$denotes the spatial correlation coefficient, $\:\phi\:\:$and $\:\delta\:$ is the spatial lag to be estimated coefficients of the policy effects and control variables, and the other parameter definitions are consistent with Eq. (1).

### Description of variables

#### Explained variables

The basic object of this paper is the carbon emissions from the power industry. Since the carbon emissions from the power industry cannot be obtained directly, they need to be obtained through the method of carbon emission accounting. This paper selects the IPCC carbon inventory method and combines it with the published relevant parameters to calculate the carbon emissions of the power industry, using the energy consumption of raw coal, coke, crude oil, gasoline, kerosene, diesel fuel, fuel oil, and natural gas to get the total amount of carbon dioxide emissions of electric power, and the data of the energy consumption comes from the China Energy Statistical Yearbook, and the specific calculation formulas are as in Eq. (3):

3 $$\:\text{C}={\sum\:}_{\text{i}=1}^{8}{\text{E}}_{\text{i}}\ast\:{\text{N}\text{C}\text{V}}_{\text{i}}\ast\:{\text{C}\text{E}\text{F}}_{\text{i}}\ast\:{\text{C}\text{O}\text{F}}_{\text{i}}\bullet\:\frac{44}{12}$$

C denotes the carbon emission of the electric power industry; E_*i*_ denotes the consumption of the ith energy source; NCV_*i*_ denotes the average low-level heat generation of the energy source corresponding to the ith energy source; CEF_*i*_ denotes the carbon emission coefficient per unit of heat of the ith energy source; COFi denotes the carbon oxidation factor of the ith energy source; and 44 and 12 are the carbon dioxide and the molecular weight of carbon, respectively.

#### Core explanatory variables

In this paper, the implementation of a carbon emissions trading market in each region is regarded as a quasi-natural experiment, and whether or not each region trades in the carbon market in that year is taken as the core explanatory variable, i.e., DID_*it*_. The key to this variable is the grouping of the samples, and the seven pilot provinces that launched the carbon emissions trading market are divided into the experimental group, and the rest of the nonpilot provinces are the control group, and this paper states that if province *i* is set up as a pilot for carbon trading in year *t*, the dummy variable of the province takes the value of 1; otherwise, it takes the value of 0. When the region *i* is Beijing, Shanghai, Tianjin, or Guangdong and *t* ≥ 2013, or when the region *i* is Hubei and Chongqing and *t* ≥ 2014, or when the region *i* is Fujian and *t* ≥ 2017, DID = 1; otherwise, DID = 0.

#### Control variables and other variables

This paper selects the following control variables that may affect regional electricity carbon emissions: population size (PZ), degree of importance of industrial pollution (DP), degree of openness (DO), energy structure (ES), energy intensity (EI), and power generation structure (PS).

This study selects the degree of government intervention (DG), electricity energy consumption (EC), and R&D innovation intensity (R&D) as mechanism variables to explore the influence mechanism of the carbon trading market and electricity carbon emissions. The specific index interpretation is shown in Table 1:

**Table 1:** Variable description.

Variable type	variable name	Variable definition	Data sources
Explained variables	Electricity CO_2_ emissions	CO_2_=$\:\sum\:{E}_{i}\ast\:{NCV}_{i}\ast\:{CEF}_{i}$	Energy data from the China Energy Statistical Yearbook.
Explanatory variables	Pilot carbon market policy	Dummy variable (0, 1)	The notification of formal establishment comes from provincial and municipal government reports
Control variables	PZ	Population of the region at the end of the year	The “China Statistical Yearbook” and the provincial and municipal statistical yearbooks
	DP	Investment in industrial pollution control/industrial value added	
	DO	Local trade in imports and exports of goods/GDP	
	ES	Coal consumption/total energy consumption	China Energy Statistical Yearbook.
	EI	Total energy consumption/GDP	
	PS	Thermal power production/generation	China Electric Power Yearbook
Mechanism variables	EC	Standard coal consumption for power generation	
	R&D	Internal expenditure on R&D/GDP	The “China Statistical Yearbook” and the provincial and municipal statistical yearbooks
	DG	General public budget expenditure/GDP	

### Data sources and descriptive notes

#### Data sources

In view of the availability and completeness of data, this paper uses the panel data of 30 provinces (autonomous regions and municipalities) in China (excluding Tibet and Hong Kong, Macao, and Taiwan) from 2003 to 2020 for the study. The data on population size, the degree of importance attached to industrial pollution, the degree of openness, the degree of government intervention, and the intensity of R&D and innovation come from the China Statistical Yearbook and the provincial and municipal statistical yearbooks; the data on various types of energy come from the China Energy Statistical Yearbook; and the data on power generation structure and power energy consumption come from the China Electric Power Yearbook. For individual missing data, the use of the linear interpolation method to supplement.

####  Descriptive statistics

Table 2 and 3 are descriptive statistics of the relevant variables, respectively. The average value of carbon emissions from electricity in each Province is increased after the implementation of the policy, but the growth rate is slowed down, the average growth rate of carbon emissions from electricity in the pilot Provinces is 21.92%. The average growth rate of carbon emissions from electricity in non-pilot Provinces is 44.88%, which is more than twice as much as the difference.

**Table 2:** Descriptive statistics of variables in pilot provinces.

Variable	Before	Mean	Standard deviation	After	Mean	Standard deviation	Difference in means
Electricity CO_2_ emissions	80	6411.9877	5259.0192	46	7817.3056	6600.6200	1405.3179
PZ	80	3897.5800	2802.5160	46	4491.5700	3542.7400	593.9900
EC	80	313.5130	25.7122	46	275.241	30.6309	− 38.2720
R&D	80	0.0208	0.0172	46	0.0280	0.0173	0.0072
DO	80	0.1006	0.0722	46	0.0735	0.0520	− 0.0271
DG	80	0.1512	0.0395	46	0.1990	0.0372	0.0478
EI	80	0.8392	0.2953	46	0.4028	0.1064	− 0.4364
PS	80	0.7778	0.2114	46	0.7661	0.2130	− 0.0117
ES	80	52.0980	11.9207	46	36.6411	15.6516	− 15.4569
DP	80	0.0033	0.0023	46	0.0017	0.0013	−0.0016

**Table 3:** Descriptive statistics of variables in non-pilot provinces.

Variable	Before	Mean	Standard deviation	After	Mean	Standard deviation	Difference in means
Electricity CO_2_ emissions	253	9342.5644	7429.7224	161	13,535.7408	11,012.2370	4193.1764
PZ	253	4526.6500	2562.8980	161	4692.1900	2704.0380	165.5400
EC	253	333.3870	30.3386	161	296.3650	11.2452	−37.0220
R&D	253	0.0098	0.0053	161	0.0136	0.0065	0.0038
DO	253	0.0294	0.0285	161	0.0246	0.0203	−0.0048
DG	253	0.2042	0.0906	161	0.2759	0.1103	0.0717
EI	253	1.4462	0.8224	161	0.8138	0.4341	−0.6324
PS	253	0.7895	0.2206	161	0.7020	0.2559	−0.0875
ES	253	75.0845	24.8429	161	72.4909	32.4952	−2.5936
DP	253	0.0046	0.0036	161	0.0035	0.0034	-0.0011

## Empirical results

### Primary regression findings

Based on the model construction and variable selection in the previous section, this section examines the effectiveness of carbon market policies on carbon emission reduction in electricity. Table 4 shows the regression results, the results are added province fixed effects and time fixed effects. The value of DID measures the effect of carbon market policies on carbon emissions in each province. Column (1) does not add control variables, the estimated coefficient of the core explanatory variable DID is negative at the 5% level of significance, -2658.7. In order to further alleviate the problem of omitted variables, column (2) adds control variables, the estimated coefficient value of the core explanatory variable DID decreases, and the level of statistical significance is strengthened to reach 1%, which is -2752.6. Regardless of the addition of the control variable or not, the estimated coefficient value of DID is significantly negative, which indicates that the carbon market policy significantly suppressed the carbon emissions of the electric power in the pilot provinces and cities, and that the carbon emissions of electric power in pilot provinces and cities decreased by about 27.53 million tons after the implementation of the carbon market policy. In summary, hypothesis 1 is fully verified.

**Table 4:** Benchmark regression results.

Variant	CO_2_	CO_2_
	(1)	(2)
$\:DID$	− 2658.694** (− 2.02)	− 2752.59*** (− 6.16)
Control variables	No	Yes
Province fixed effects	Yes	Yes
Year fixed effects	Yes	Yes
Sample size	540	540
R^2^	0.8734	0.9070

*, **, and *** denote significance levels of 10%, 5%, and 1%, respectively, with t-values in parentheses. The table below is the same.

### Parallel trend test (dynamic effect test)

1. Event study diagram.

When using the double difference model to assess the effectiveness of the policy’s impact on the event, the key premise is that the experimental group and the control group should have a parallel trend of change before the implementation of the policy. Therefore, in order to examine the effect of the dynamic treatment of the carbon market pilot policy over time and to verify the assumption of a parallel trend of the double difference method, it is necessary to judge that, after the implementation of the policy, the change in the trend of carbon emissions from electricity in the pilot provinces and municipalities was basically caused by the carbon market policy, rather than being difficult to observe. This paper draws on the research framework of McGavock and adopts the event study method to test the parallel trend^36^; the regression model is as in Eq. (4):

4 $$\:{y}_{i,t}=\alpha\:+{\sum\:}_{k=-14}^{6}{\beta\:}^{k}DI{D}_{i,t}^{k}+\sum\:\gamma\:{X}_{i,t}+{\mu\:}_{i}+{\lambda\:}_{t}+{\epsilon\:}_{i,t}$$

where is the policy variable relative to the kth year generated by taking the year of implementation of the policy in province i as the 0th period as a reference, and its value rule is: when province i in year t is in the kth year before/after the time of its pilot, it is taken as 1; otherwise, it is taken as 0. In this paper, the year before the implementation of the pilot is taken as the base year for the event analysis, and it is the coefficient of the effect of the policy relative to the kth year, which measures the differences in electricity carbon emission reduction between provinces implementing the carbon market pilot and provinces without launching the carbon market in different years. If the coefficient is not statistically significant at k < 0, it indicates that there is no significant difference in electricity carbon emissions between the provinces that implemented the carbon market pilot before implementation and those that did not, indicating that the parallel trend assumption is satisfied, and the estimation results can be plotted at 90% confidence intervals. The coefficient at j ≥ 0 measures the annual treatment effect of the pilot policy, which, if statistically significant, indicates that the implementation of the carbon market pilot had a substantial impact on electricity carbon emissions in that year. Other variable definitions remain consistent with the baseline regression model setup.

**Fig. 2:**
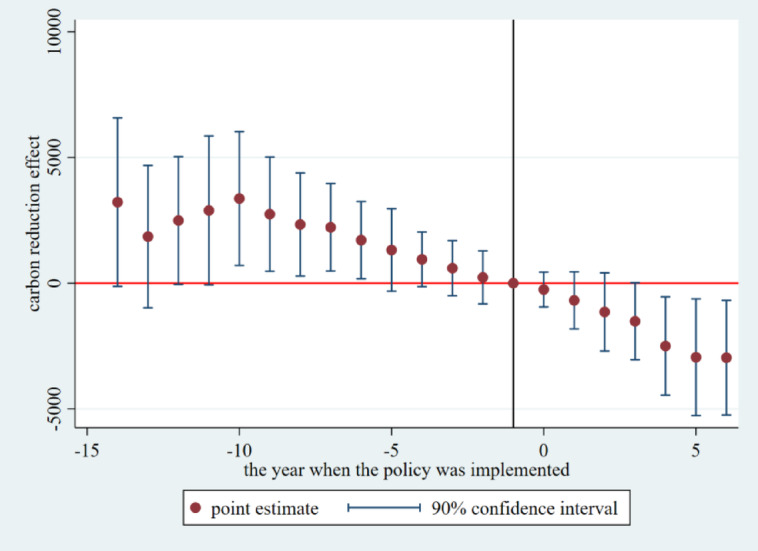
Pre-treatment trend event study.

The results, as shown in the Fig. 2, demonstrate the results of the pre-treatment trend event study of the carbon market pilot policy in the first 14 periods and the second 6 periods, the coefficients of the 14 periods before the implementation of the policy are statistically insignificant as a whole, which indicates that there is no significant difference between the electricity carbon emissions of the provinces in the treatment group and the control group before the implementation of the policy, and that the assumption of the parallel trend is valid. In addition, the dynamic effect analysis found that in the fourth year after the implementation of the policy, the carbon emission reduction effect began to appear significantly, the carbon emission reduction effect was strengthened in the fifth year, and the carbon emission reduction effect of electricity reached the maximum in the sixth year. At the same time, there is a certain lag in the carbon market policy effect, which is caused by the rigidity of the system, behavioral habits, and conversion costs, and it takes time for each subject to make decisions after receiving the policy signals.

(2) Parallel trend sensitivity test.

**Fig. 3:**
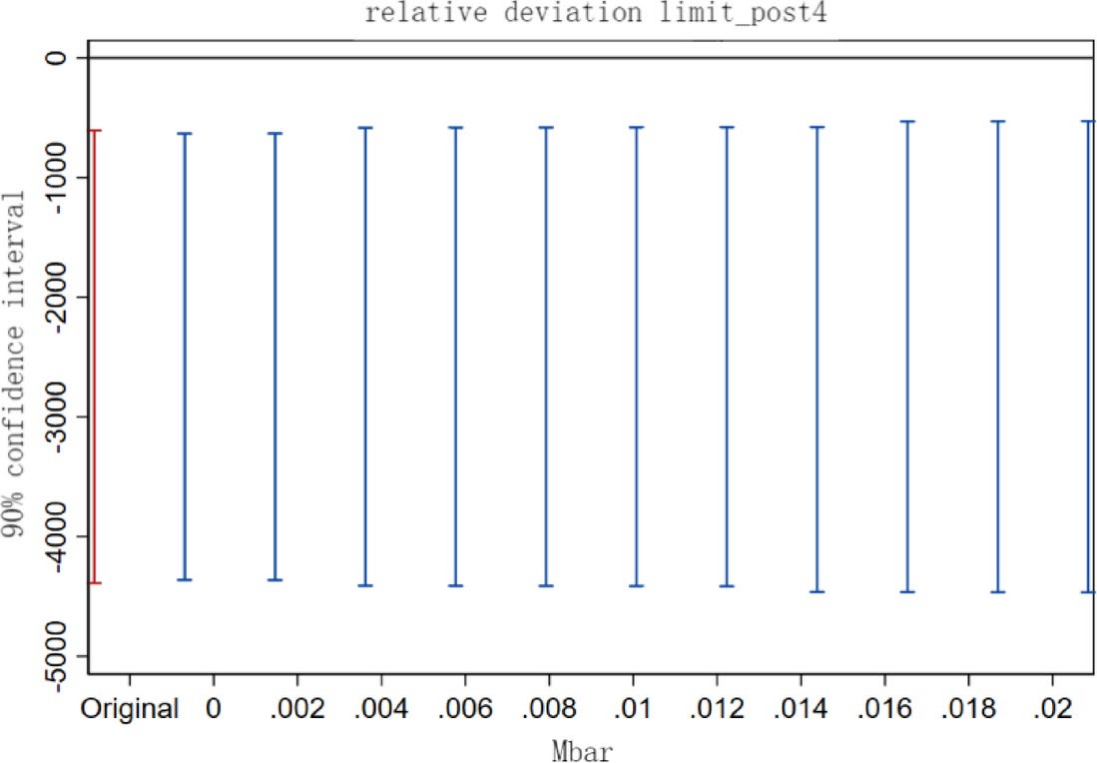
Sensitivity test for parallel trends in the fourth year after policy implementation.

**Fig. 4:**
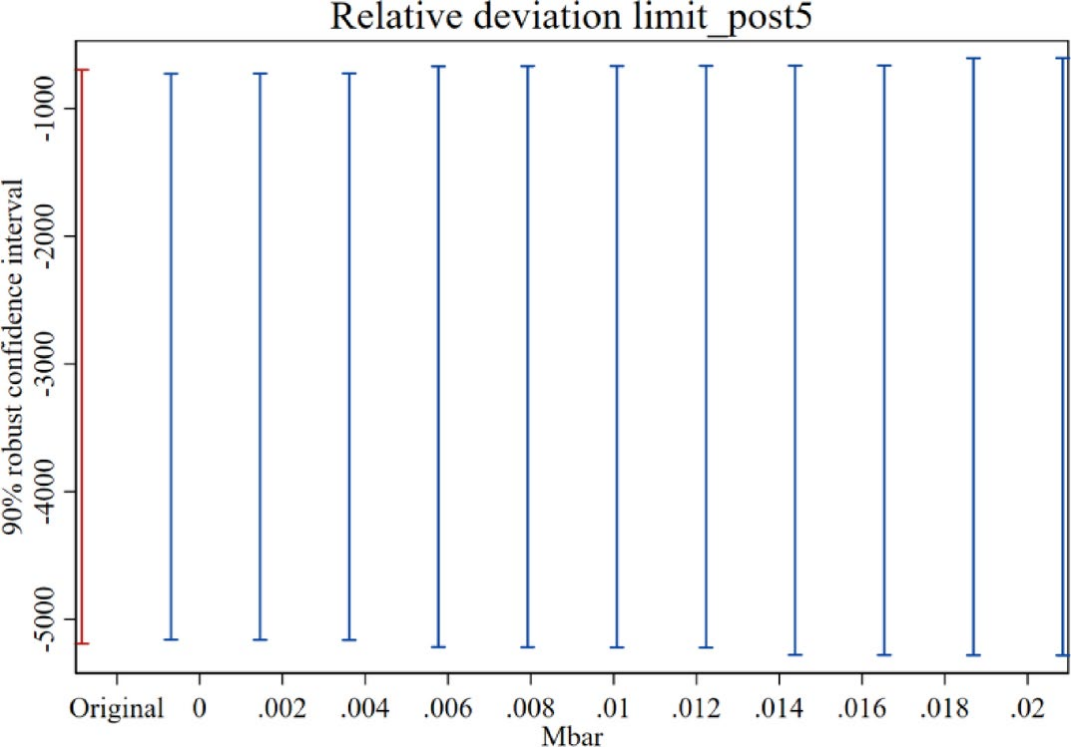
Sensitivity test for parallel trends in the fifth year after policy implementation.

**Fig. 5:**
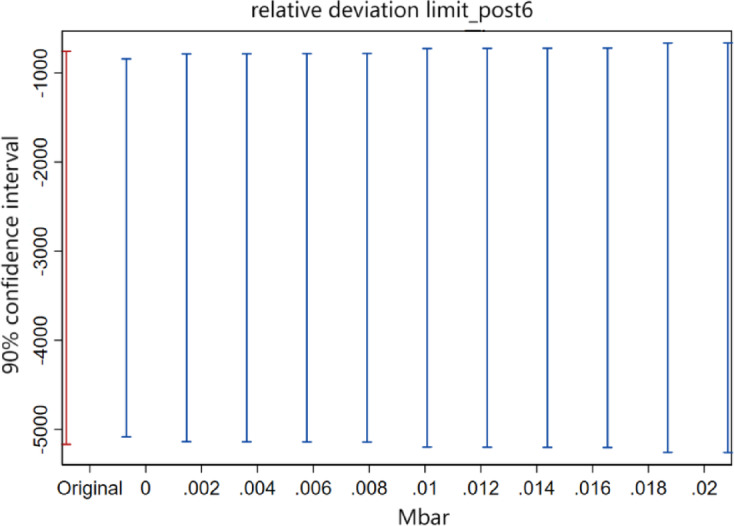
Sensitivity test for parallel trends in the sixth year after policy implementation.

Some scholars have found that the pre-treatment trend test does not serve as empirical evidence that the parallel trend hypothesis works^37^, and the traditional pre-treatment trend test may cause bias and distortion in estimation and inference. In order to solve this problem, we refer to the practice of Biasi et al.^38^, sets the maximum degree of deviation Mbar = 1×standard error, and at the same time constructs the confidence interval that corresponds to the above-mentioned deviation degree that corresponds to the confidence interval of the post-treatment point estimate in order to test the sensitivity of the parallel trend of the treatment effect after the implementation of the carbon emissions trading policy. If the confidence interval of the post-treatment point estimate does not contain a value of 0, it indicates that the treatment effect has a good robustness to the degree of deviation from the parallel trend. The results (Figs. 3, 4 and 5) show that the carbon reduction effect after the fourth year of policy implementation is still very robust under the set limit on the relative degree of deviation. The test results indicate that even if there is a certain degree of deviation from the parallel trend, the carbon emissions trading policy still has a significant electricity emission reduction effect on the pilot region.

### Tests for heterogeneous treatment effects

When there is group and time heterogeneity in the treatment effects, even if the parallel trend assumption is satisfied, the estimates of the treatment effects are biased. Based on the multi-period, multi-individual multiplicative scores model and the corresponding estimator (DID_M_) proposed by de Chaisemartin et al.^39,40^, a diagnosis of possible heterogeneous treatment effects in the baseline regression is made. The carbon emissions trading policy was implemented in 2014 and 2017, and there is temporal heterogeneity, so negative weight decomposition is carried out to analyze the severity of the bias due to temporal heterogeneity; there is also the possibility of bias in the treatment results due to the existence of heterogeneity among provinces, so the DID_M_ estimator is used to conduct a robustness test.

1. Negative weight decomposition.

After the negative weight decomposition, as shown in Table 5, the coefficients obtained from the resultant benchmark model are the weighted sum of 46 pairs of 2 × 2 double difference estimators, of which only one pair has a negative weight of − 0.0007, which has a minimal impact on the final results, and it can be assumed that the time heterogeneity hardly causes the coefficients obtained from the benchmark model to be biased.

**Table 5:** Negative weights decompose the results.

Treat. var: did	#ATTs	∑weights
Positive weights	45	1.0007
Negative weights	1	− 0.0007
Total	46	1.0000

(2) DID_M_ estimator.

**Fig. 6:**
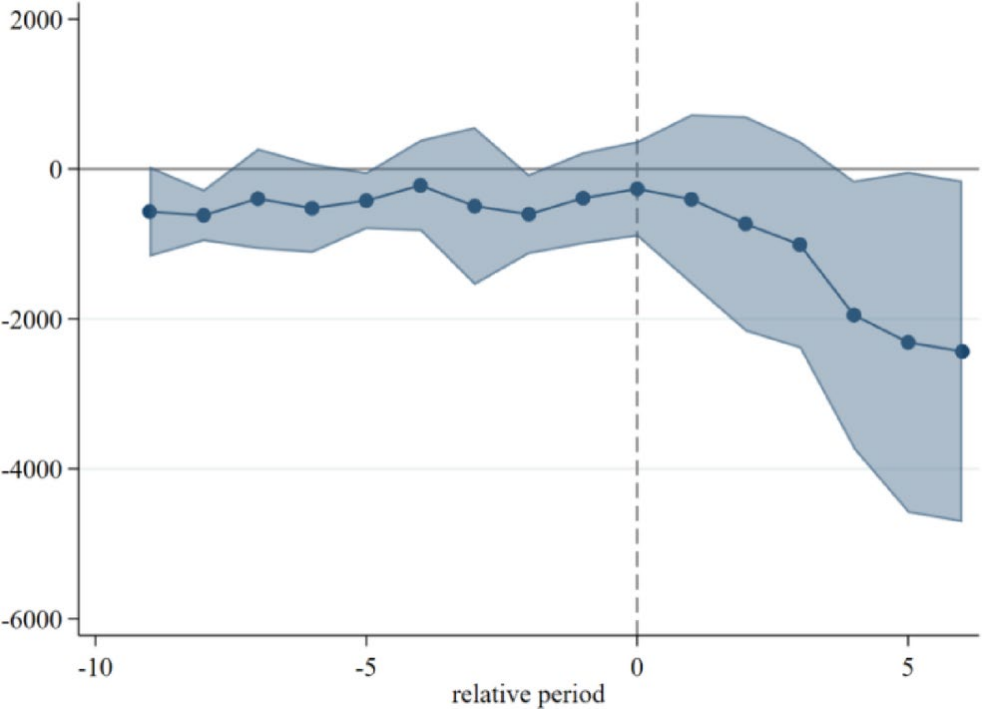
Map of event studies based on the de Chaisemartin, C. et al. methodology.

As can be seen from Fig. 6., incorporating the possibility that heterogeneity across provinces can lead to biased estimates, before the implementation of the carbon emissions trading pilot policy, the trend of carbon emission changes in the treatment and control groups is roughly the same, which fulfills the assumption of parallel trends; and after the fourth period after the implementation of the carbon emissions trading market policy, the carbon reduction effect of the policy begins to appear, confirming the validity of the regression results of the baseline model.

### Endogenous treatment

**Fig. 7:**
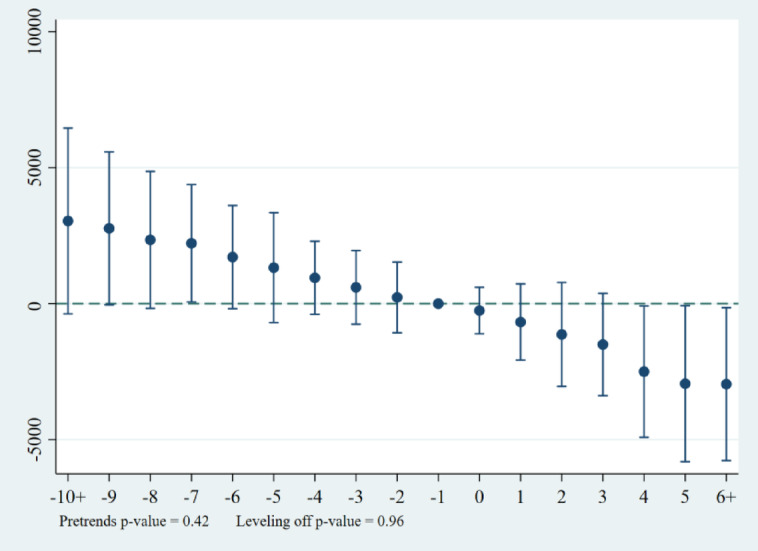
Map of event studies based on the Freyaldenhoven et al. methodology.

Considering that there May be unobservable confounding variables related to the policy variables and outcome variables at the same time, i.e., there is the problem of endogeneity that causes the outcome variables to show ex ante trends, which leads to the failure of the parallel trend hypothesis, the method proposed by Freyaldenhoven et al.^41^ is used for the treatment of ex ante trends. (Fig. 7). It can be seen that the p-value of the ex ante trend test is 0.46, which is greater than 0.1, indicating that the original hypothesis of “there is no pre-treatment trend” cannot be rejected, which means that the probability of the existence of an ex ante trend before the implementation of the policy is very small; the p-value of the corrected multiple tests is 0.96, which is greater than 0.1, indicating that the original hypothesis of “including all dynamic effects” cannot be rejected, which supports the results of the ex ante trend test. These tests further demonstrate the robustness of the results of the benchmark regression model

### Robustness test

1. Placebo test.

In order to prevent the experimental and control groupings from being potentially affected by other random factors, the method of Bai et al.^42^ is adopted to conduct a placebo test to enhance the robustness of the results in this paper. This is done by randomly selecting the same number of samples as the original treatment group from all samples and randomly generating the time of policy implementation to construct a new double randomized treatment group with both province and policy time randomized. Based on this, the baseline regression model is re-estimated, and the experiment is randomly repeated 500 times.

**Fig. 8:**
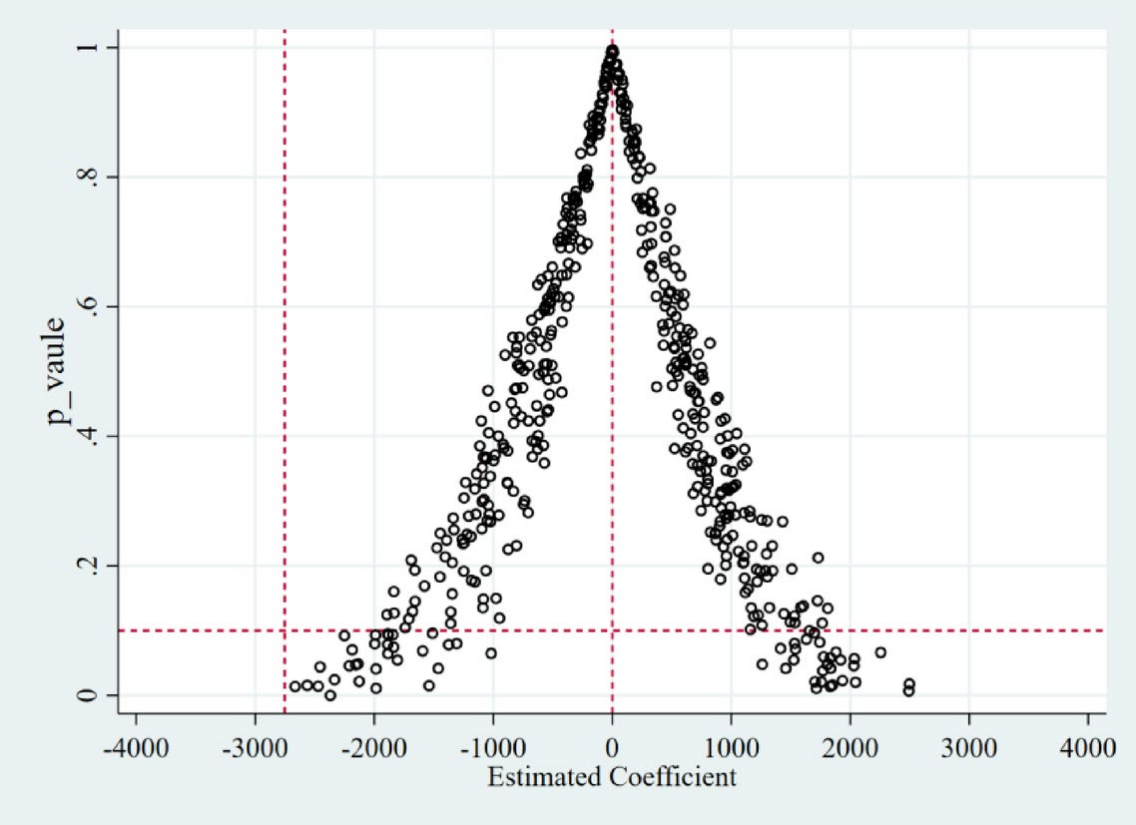
Placebo test.

As shown in Fig. 8, the estimated coefficients obtained from random sampling are distributed around 0 and normally distributed, and almost no estimated coefficients fall on the right side of the regression coefficient of the benchmark model (− 2752.596), indicating that unobservable shocks or random factors do not significantly promote carbon emission reduction of enterprises and that the reduction of carbon emissions at the provincial level is indeed due to the implementation of the carbon emissions trading policy.

(2) Counterfactual estimation.

**Fig. 9:**
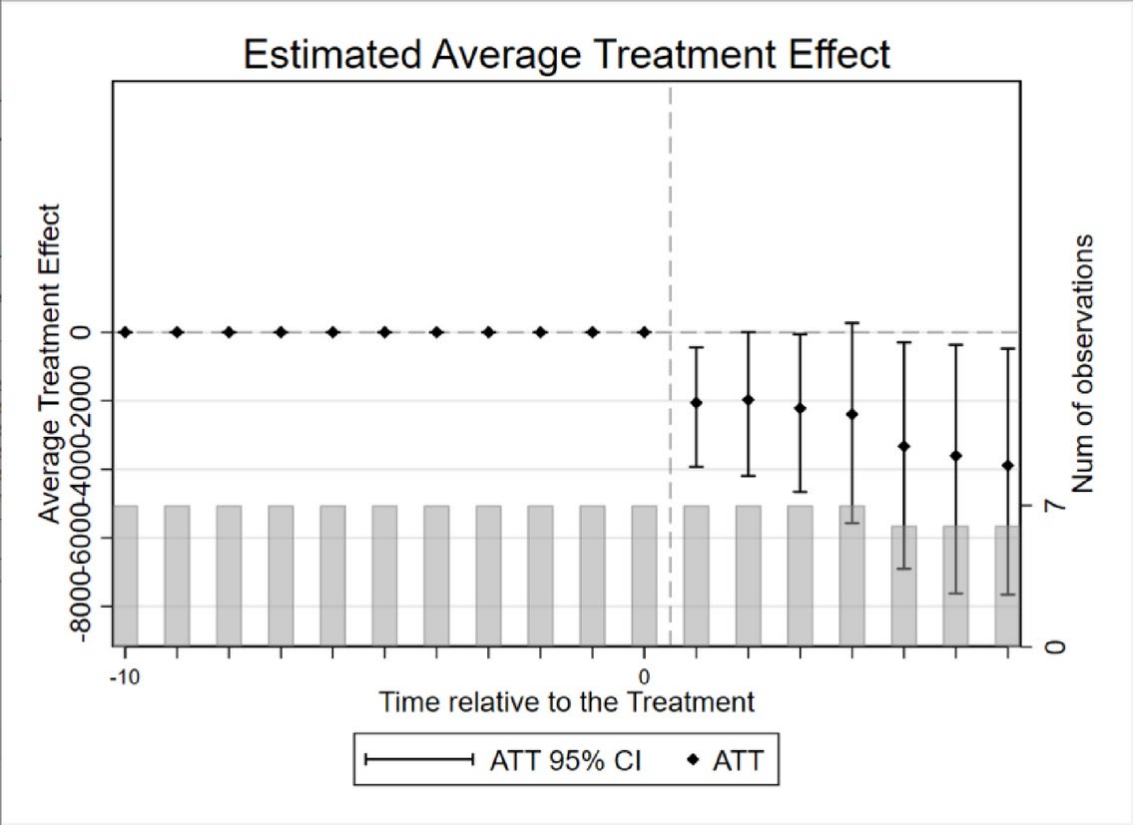
Diagram of event study based on Liu et al.‘s approach.

To further exclude the possibility of bias in the assessment of policy effects obtained from the baseline regression model in the presence of heterogeneity in treatment effects or the presence of unobserved time-varying confounders, based on the counterfactual estimation framework of causal effects proposed by Liu et al.^43^, a matrix completion estimator (Matrix Completion Estimator (MC)) is used for interpolation, and the results are shown in Fig. 9. It can be seen that the regression results of the benchmark regression model remain robust even by directly estimating the counterfactual results, excluding the effects of heterogeneity in treatment effects or unobserved time-varying confounders.

### Spatial spillover effects

**Table 6:** Spatial Durbin model regression result.

	W1	W2	W3
	CO_2_	CO_2_	CO_2_
DID	− 2770.553*** (701.858)	− 5818.026*** (934.944)	− 5817.701*** (934.978)
Wx: DID	1501.853 (2771.936)	6245.200*** (1618.357)	6244.943*** (1618.716)
Direct	− 2733.605*** (731.653)	− 5567.092*** (922.525)	− 5566.88*** (922.572)
Indirect	942.435 (4004.564)	6107.393*** (2001.932)	6107.248*** (2002.442)
Total	− 1791.169 (4223.725)	540.302 (1708.886)	540.368 (1709.280)
ρ (Spatial: rho)	0.300 (0.118)	0.278*** (0.074)	0.278*** (0.074)
R-squared	0.566	0.624	0.624

We adopt the spatial Durbin model to examine the spatial spillover effects of the pilot carbon market policy. To ensure the robustness of the empirical test results, the inverse distance weight matrix W1, the economic distance weight matrix W2, and the economic-geographical nested weight matrix W3 are used to conduct the tests, and the tests under the three spatial weight matrices are reported in Table 6. The spatial correlation coefficients ρ of W2 and W3 in Table 6 show statistically significant positive values, indicating a positive correlation between CO_2_ emissions among different provinces and cities. Second, the estimated coefficients of Wx: DID also show statistically significant positive values, which initially support the positive spatial spillover effect of the pilot carbon market policy.

In order to correctly identify the spillover effect of the pilot carbon market policy on electricity carbon emission reduction, autoregressive partial differential processing is further adopted to decompose its spatial effect into direct effect, indirect effect, and total effect, and the results are shown in Table 6. Under the three kinds of weighting matrices, the direct effect of the pilot carbon market policy is significantly negative, indicating that in the areas where the policy has been implemented, it effectively exerts the effect of electricity carbon emission reduction, which further provides a further This further provides a robustness test for the electricity carbon emission reduction effect of the pilot carbon market policy. In the spillover effect, the coefficients of W2 and W3 are significantly positive, indicating that there is a certain emission transfer effect between neighboring regions where the carbon policy is implemented. It is worth noting that the results obtained by W2 and W3 are more significant than those of W1, indicating that the explanatory power of pure geographic proximity to the diffusion of the policy is limited, and that W2 and W3 better reflect the diffusion effect of the policy among economically linked regions. Economic linkages produced positive spatial spillovers that may lead to increased emissions in other regions, and the overall effect is therefore not significant. This emphasizes the need to consider cross-regional synergistic mechanisms when formulating regional emission reduction policies to avoid carbon leakage and to strengthen interregional cooperation to achieve overall emission reduction targets.

## Mechanism analysis

In order to further analyze the channels through which the carbon trading policy affects the carbon emissions of electricity, this paper examines the mechanism through which the carbon trading policy realizes the mitigation of the growth of the carbon emissions of electricity in terms of the degree of government intervention, the energy consumption of electricity, and the intensity of R&D and innovation. The ratio of general public budget expenditures to gross regional product (GDP) is used to represent the degree of government intervention, the standard coal consumption of electricity generation is used to represent the energy consumption of electricity, and the ratio of internal expenditures on R&D to GDP is used to represent the intensity of R&D and innovation.

### Intensity of government intervention

The intensity of government control exercised on the market can effectively enhance the carbon emission reduction effect of the carbon market^11^.Administrative intervention is an important tool to compensate for the loss of operational efficiency of the carbon market. When the development of the carbon market is low, the local government, facing the pressure of carbon emission reduction, will be enhanced through the control to urge the main body of the control of emissions to fulfill its obligations (i.e., to ensure that there is no untraded excess carbon emissions). 7 pilot regions of the carbon market started late, the implementation year is short, and the construction of the relevant market mechanism is still in a number of imperfections; therefore, the carbon market is still in the early stage of development. It is necessary to rely on administrative intervention to realize carbon emission reduction. The carbon market still needs to rely on administrative intervention to realize carbon emission reduction. The government needs to invest resources in the infrastructure of the carbon market, intervene in the organization of power market transactions, price behavior, etc., to expand the coverage of the power generation industry, and improve the influence of the market. With the gradual improvement of the carbon market system construction in the pilot regions and the gradual development of the national carbon market, it is expected that the carbon emission reduction effect of carbon trading will be gradually enhanced in the future. The results of the benchmark regression of carbon market policy on policy intervention are shown in column (1) of Table 7, and the DID coefficient is positive and significant at the 1% level, reflecting the positive influence of carbon market trading policy on the degree of government intervention, which indicates that the carbon market pushes the government to flow more capital investment to energy-saving and carbon reduction projects to promote carbon emission reduction of the power industry. Hypothesis 2 is fully tested.

### Power energy consumption

In the study of the driving force of carbon emissions in the power industry, the rate of coal consumption in power generation plays a major inhibiting role^44^; the carbon market policy makes the cost of carbon emissions of power enterprises visible by setting a price for carbon emissions. For thermal power enterprises, the carbon emissions in the power generation process are closely related to the coal consumption of power generation, and the increase of carbon cost directly raises the marginal cost of power generation, and power enterprises will take corresponding measures to optimize the coal consumption of power generation in order to reduce the overall cost of power generation. Under the synergistic effect of the carbon market and the power market, the competitiveness of high-efficiency, low-emission generating units in the market has been enhanced^45^, which prompts thermal power enterprises to improve their market competitiveness by reducing the coal consumption of power generation in order to avoid being replaced by cleaner power generation methods. Carbon market policy also promotes the optimization of power structure^46^; thermal power enterprises under the pressure of carbon cost will reduce the power generation capacity of high-carbon units and increase the proportion of low-carbon units, which not only reduces the overall coal consumption of power generation but also achieves the goal of carbon emission reduction in power. The regression results of the carbon market on power energy consumption are shown in column (2) of Table 7. The coefficient of power energy consumption is negative and significant at the 5% level, which indicates that after the implementation of the carbon trading policy, the power energy consumption is significantly reduced by about 12.5 g of coal per kilowatt hour, and the efficiency of power generation is improved. Hypothesis 3 is fully tested.

### R&D innovation intensity

Technological innovation plays a significant promoting effect on regional pollution reduction and carbon management^47^. According to Porter’s hypothesis, appropriate environmental regulations can incentivize enterprises to increase innovation investment, improve productivity, and offset the cost of environmental protection^48^.Strict carbon emission quotas and carbon pricing mechanisms increase the cost of carbon emissions, and this cost-driven mechanism forces power companies to reduce carbon emissions through technological innovation and management optimization, thus reducing the need to purchase carbon quotas. When the volatility of the carbon cost is large, the size of the cost and benefit will motivate the power enterprises to choose the long-term benefit and increase the investment in energy-saving technology innovation and R&D to reduce the carbon emissions of the power industry. Table 7 column (3) results can be obtained; the coefficient of R&D innovation intensity is negative and significant at the 5% level, which indicates that the carbon market policy inhibits R&D innovation to a certain extent, and the instability of carbon quota prices will weaken the confidence of enterprises in long-term R&D to a certain extent^49^. The carbon market policy forces enterprises to carry out technological R&D, but the cost of technology needs to be accumulated^50^, and if it exceeds the cost of quota purchase, enterprises will choose to buy a quota in the short-term strategy to cope with it, and the cost of carbon quotas may crowd out the investment for R&D^51^, which from the side of the intensity of China’s carbon trading market still needs to be strengthened to further develop the carbon price. Further improve the development of carbon pricing, increase R&D investment, and incentivize the research and development of carbon-reducing technologies. Hypothesis 4 is fully tested.

**Table 7:** Mechanism analysis results.

Variant	(1)	(2)	(3)
	DG	EC	*R*&D
DiD	0.042*** (4.94)	− 12.536** (− 1.90)	− 0.003** (− 2.26)
Control variables	Yes	Yes	Yes
Year fixed effects	Yes	Yes	Yes
Province fixed effects	Yes	Yes	Yes
Sample size	540	540	540
*R* ^2^	0.6463	0.2613	0.7875

## Heterogeneity analysis

###  Heterogeneity in the degree of importance attached to environmental governance

Regions that emphasize environmental governance will set clear carbon emission reduction targets and incorporate them into the assessment systems of local governments and enterprises. In regions that attach high importance to environmental governance, the government and enterprises will provide sufficient financial guarantees for the implementation of the policy, and the government will introduce a series of policies and measures to support environmental governance, such as financial subsidies, tax incentives, and green finance. These policies can reduce the cost of emission reduction and increase the enthusiasm of enterprises to participate in environmental governance. On the contrary, in regions where low importance is attached to environmental governance, the industrial structure is more irrational, there are more heavily polluting and high-emission enterprises, the environmental pressure is greater, and the implementation of the policy is more incomplete, the governance results are not good.

Based on the data of the elasticity coefficient of investment in environmental pollution control (elasticity coefficient of investment in environmental pollution control = investment growth rate in environmental pollution control/GDP growth rate)^52^ of each region of China from one year before the implementation of the pilot program to 2012, we divided the regions into two groups of high environmental importance group (coefficient value is greater than or equal to 1, i.e., the growth rate of investment in environmental pollution control is faster than or equal to the GDP growth rate) and low environmental importance group (coefficient value is less than 1, i.e., the growth rate of investment in environmental pollution control is slower than the GDP growth rate). The regression results are shown in columns (1) and (2) of Table 8, which show that the role of pilot carbon market policy is not significant in the regions with high emphasis on environmental governance, while the role of pilot carbon market policy is significant at 1% level in the regions with low emphasis on environmental governance, which may be due to the fact that in the regions with high emphasis on environmental governance, some environmental policies have been given the opportunity to be tried out first in these regions, and their environmental policies have been more perfect, so the carbon market policy plays a role relatively perfect, so the role of carbon market policy is not significant, while in the low importance of environmental policy in the region, its environmental governance system is not yet complete, the policy implementation and supervision is not in place. Carbon market policies use market-based economic means to internalize the cost of its carbon emissions, giving enterprises cost pressure^30,53^ and flexibly regulating the distribution relationship between the government and power companies to play the effect of emission reduction.

### Regional heterogeneity

The previous section has estimated the overall impact of the pilot carbon market policy on electricity carbon emission reduction in an average sense. Considering that China’s geographic region is relatively vast, there are large differences in the level of economic development, energy structure, and the level of low-carbon technology in different regions, and the role played by the pilot carbon market policy on them will also be slightly different. Generally speaking, areas with higher level of economic development, higher degree of marketization, more sensitive to the implementation of the carbon market, faster into the construction, while the region can provide technical and financial support for the construction of the carbon market to promote the application of new energy power generation, power enterprises, system transformation, etc. China’s complex geographic features determine the uneven distribution of renewable energy resources, such as hydro, wind and solar energy, showing a distribution pattern of “more in the north, less in the south, more in the west and less in the east. In regions with relatively diversified energy structures, the proportion of clean energy power generation in energy consumption is gradually increasing, and the effect of carbon emission reduction from electricity should be more obvious. In regions with a higher technological level, the power generation technology is cleaner and more efficient, and the production efficiency is improved, which affects the carbon emissions of the power industry in the region.

In order to deepen the understanding of the carbon emission reduction effect of the pilot carbon market policy on electricity, it is necessary to further examine the regional heterogeneity of the impact of the pilot carbon market policy on electricity carbon emissions. Based on the basic database of Beijing’s macroeconomic and social development and the geographic location differences that divide China’s regions into East, Central and West, this paper introduces the interaction term of the region where the pilot carbon market is located with the disposal variable in the baseline regression model to test whether there is regional heterogeneity in the carbon emission reduction effect of the pilot carbon market policy on electricity. Table 8’s (3), (4), and (5) columns show the regression results of the heterogeneity analysis grouping. Regardless of the eastern, central, and western regions, the DID coefficients are significantly negative, which is consistent with the previous results, but the coefficients differ in magnitude, which suggests that there is a difference in the carbon emission reduction effect of the carbon markets in the eastern, central, and western regions on the electric power industry. For the eastern region, the DID coefficient is the smallest, -3069.42, and significant at the 1% level, indicating that after the implementation of the carbon market policy, the carbon emissions from electricity in the pilot provinces and cities in the eastern region have been reduced by about 30,694,200 tons.

**Table 8:** Results of heterogeneity analysis.

variant	HG	LG	Eastern	Central	Western
DID	− 414.5952	− 4119.809***	− 3069.425***	− 1377.248**	− 1778.517***
Control variables	Yes	Yes	Yes	Yes	Yes
Year fixed effects	Yes	Yes	Yes	Yes	Yes
Province fixed effects	Yes	Yes	Yes	Yes	Yes
R^2^	0.9044	0.9474	0.9549	0.8993	0.9109
Sample size	378	288	252	270	270

## Conclusion and policy implications

Based on the panel data of 30 provinces (cities) in China from 2003 to 2020, this paper empirically evaluates the emission reduction effect of the carbon trading policy by using the multi-period multiplier method and utilizes a variety of parallel trend sensitivity tests and heterogeneity treatment effect tests and other robustness tests. The spatial spillover effects of pilot carbon market policies were tested using the spatial Durbin model to make the policy assessment results more accurate. On this basis, the mechanism test is used to further discuss the mechanism of government intervention degree, electricity energy consumption, and R&D and innovation intensity in the emission reduction effect of carbon trading policies, while the heterogeneity analysis is conducted to understand the characteristics of the pilot carbon market policies under different degrees of emphasis on environmental governance and regional characteristics.

The main conclusions of this paper are: (1) Carbon trading policy can effectively slow down the intensity of regional electric power carbon emissions and has a significant emission reduction effect, which still holds after parallel trend tests, placebo tests, and other robustness tests. It is important to note that the impact of China’s carbon emissions trading mechanism on the pilot provinces may be overestimated due to certain spillover effects when the mechanism is implemented in individual pilot regions rather than on a national scale; (2) Carbon market policies have positive spatial spillover effects through economic linkages, and there is some displacement of carbon emissions; (3) The emission reduction effect of the carbon trading market is mainly realized through government intervention and reduction of electric power energy consumption, but it does not strengthen the intensity of research and development and innovation up to 2020. The coefficient of R&D innovation intensity is negative, which has not effectively promoted technological innovation for emission reduction. (4) The impact of the carbon market on electricity carbon emissions in different regions is heterogeneous, in which the carbon emission reduction effect of electricity in the eastern region is stronger than that in the central and western regions, and carbon markets are more effective in areas where environmental governance is a low priority.

Based on the above conclusions, this paper puts forward the following suggestions:

First, from the viewpoint of the carbon reduction effect in the pilot provinces, the market-incentivized carbon emissions trading slows down the growth of carbon emissions from electricity and further improves the corresponding emission reduction measures in the carbon market for the power industry, such as the quota allocation mechanism, so as to make it more scientific and reasonable and to accurately reflect the emission reduction potentials and costs of the enterprises, so as to promote the electricity-carbon linkage, and contribute to the realization of the “dual-carbon” goal. 

Second, increase investment in technological innovation. The mechanism test in the article shows that the intensity of R&D innovation has a significant impact on the emission reduction effect of carbon emissions trading policy, but the driving force is negative. Therefore, the government should compensate enterprises for green innovation, increase support for R&D, demonstrate and promote low-carbon technologies, and focus on supporting the R&D of key technologies such as renewable energy generation and energy storage technologies. At the same time, improve the green technology innovation policy environment, such as tax incentives, financial subsidies, etc., to reduce the enterprise’s R&D costs and risks and encourage enterprises to improve their own R&D capabilities to promote the level of green low-carbon technology.

Third, the government should increase the intervention. The analysis of the mechanism of the article shows that the degree of government intervention is the most significant. The government should increase support for electric power enterprises, taking into account the differences in the level of economic development of different regions, the energy structure, and the degree of importance of environmental governance. The government should formulate differentiated carbon emission reduction policies. For regions that attach low importance to environmental governance, policy support and supervision should be increased to urge them to accelerate the process of carbon emission reduction. For the central and western regions, greater policy inclination and financial support are needed to enhance their emission reduction capacity and technology level, narrowing the gap with the eastern regions. Meanwhile, the implementation of carbon market policies should be synergized with other environmental governance policies to form a policy synergy.

Fourth, from the results of the spatial Durbin model, it can be seen that the diffusion effect of the pilot carbon market policy is better among the economically linked regions, and the government needs to set up a cross-regional collaboration framework for carbon emission reduction in electric power to avoid “beggar-thy-neighbor” competition and to shift the spillover effect from “carbon emission transfer” to “low-carbon technology diffusion” through regional joint research and development and the construction of clean energy infrastructures. 

Through the research in this paper, policymakers need to establish a carbon price stabilization mechanism to avoid drastic price fluctuations to weaken the innovation power of enterprises and can direct the carbon market proceeds to invest in the power industry’s R&D of low-carbon technologies and reduce the technological risk through the “innovation fund + tax incentives.” In the future, we need to take the carbon market as the core link, integrate technology, policy, and market forces, and build a synergistic and optimized low-carbon transformation ecology of power “emission reduction-security of supply-economy.”

## Data Availability

The data for this research result are available from the corresponding author upon reasonable request.
